# Appropriate surgical modalities for stages T2a and T2b in the eighth TNM classification of lung cancer

**DOI:** 10.1038/s41598-017-13495-w

**Published:** 2017-10-12

**Authors:** Fenglong Bie, Xiao Qu, Xudong Yang, Zhaofei Pang, Yufan Yang, Shaorui Liu, Wei Dong, Jiajun Du

**Affiliations:** 1 Institute of Oncology, Shandong Provincial Hospital Affiliated to Shandong University, 324 Jingwu Road, Jinan, 250021 P. R. China; 2 Department of Thoracic Surgery, Shandong Provincial Hospital Affiliated to Shandong University, 324 Jingwu Road, Jinan, 250021 P. R. China

## Abstract

Patients with tumors of 3 to 5 cm were divided into stages T2a (3 to 4 cm) and T2b (4 to 5 cm) based on the 8^th^ tumor-node-metastasis staging system for lung cancer. The objective of our study was to explore appropriate surgical modalities for the new stages, T2a and T2b. We selected 6,996 node-negative non-small-cell lung cancer patients with tumor sizes of 3 to 5 cm, diagnosed between 2009 and 2013, from the Surveillance, Epidemiology, and End Results (SEER) program. The Pearson ${\chi }^{2}$. statistic test and Kaplan–Meier curve were used to analyze patient data. The prognosis of patients with stage T2a was significantly better than that of patients with stage T2b, both in overall survival (p = 0.018) and lung cancer specific survival (p = 0.001). For patients with stage T2a, lobectomy had a significantly better outcome. For patients with stage T2b, surgical modalities including pneumonectomy, segmental resection and lobectomy, had similar outcomes in terms of survival. Consequently, lobectomy was the most appropriate surgical treatment modality for new stage T2a patients, whereas, for new T2b patients, treatment outcome did not vary significantly with the choice of surgical modality.

## Introduction

Lung cancer is the most commonly diagnosed cancer and remains the leading cause of cancer-related death in both males (27%) and females (25%)^1^. Non-small-cell lung cancer (NSCLC) accounts for approximately 85% of all lung cancer cases^2^. Available therapy choices for lung cancer, such as surgery, chemotherapy and radiation therapy, have little impact on patient survival, due to metastasis and recurrence^3–5^. For early-stage lung cancer, surgery is the most common treatment modality^6,7^. Therefore, accurate staging is crucial in determining the most appropriate treatment approach.

The TNM (tumor-node-metastasis) staging system, based on primary tumor size, number of lymph nodes affected, and the presence/absence of metastasis, is one of the most important lung cancer staging systems, according to the International Association for the Study of Lung Cancer (IASLC)^8^. This staging system is used for prognosis in lung cancer patients and serves as guidelines in choosing appropriate treatment methods^9–12^. The 8th edition of TNM staging for lung cancer has brought multiple changes to the 7th edition staging system. This revision was prompted by recommendations made in the IASLC International Staging Project^13,14^.

The revised staging system for lung cancer is closely associated with the choice of surgical procedure^15^. The previous NCCN Guidelines (Non-Small Cell Lung Cancer, Version 8.2017) only classed tumors as operable or inoperable based on the negative mediastinal nodes. Treatment methods for operable tumors include surgical exploration and resection, mediastinal lymph node dissection or systematic lymph node sampling^16^. However, the NCCN Guidelines do not provide specific information which would allow the selection of the most appropriate surgical approach for tumors of different sizes. Some recent studies have explored the most appropriate surgical modalities according to tumor size for NSCLC patients. For example, He *et al*. showed that lobectomy was better than sublobar resection for NSCLC patients with tumors measuring 0 to 2 cm^17^. To our knowledge, no such study has been conducted for NSCLC patients with tumors of 3 to 5 cm. Therefore, we devoted to finding the appropriate surgical modalities for these patients.

NSCLC patients with tumor size 3 to 5 cm were divided into stage T2a (3 to 4 cm) and stage T2b (4 to 5 cm) groups according to significant differences in survival in the 8th edition of the TNM classification for lung cancer^8^. Due to changes in the staging system, the most appropriate surgical modality for these two groups of NSCLC patients might differ. Consequently, the objective of our study was to identify the most appropriate surgical methods for NSCLC patients of new stages T2a and T2b.

## Results

### Baseline clinical and pathological information

**Figure 1:**
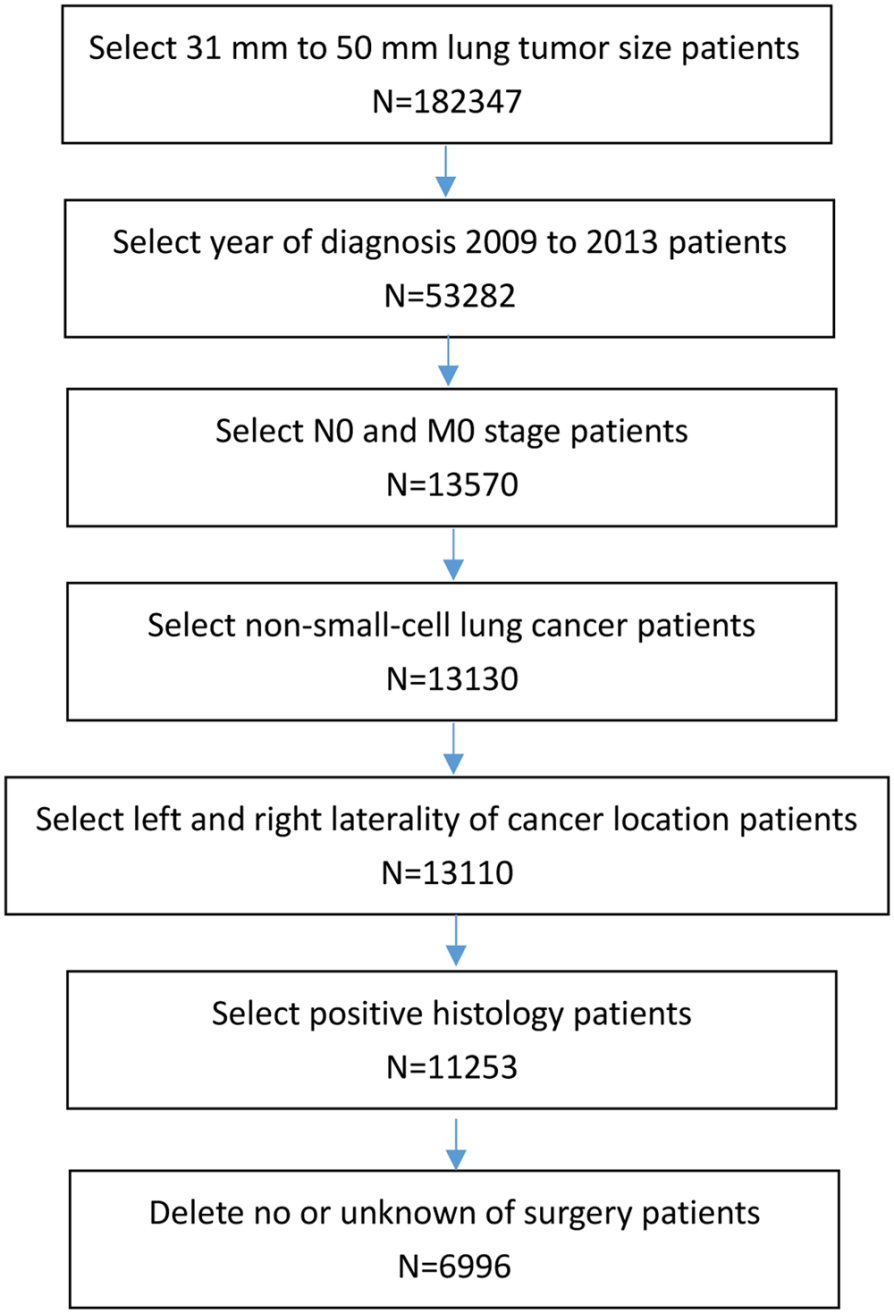
Flow sheet of eligible patients included in the study.

NSCLC patients with tumors measuring 3 to 5 cm, diagnosed between 2009 and 2013, were selected from the Surveillance, Epidemiology, and End Results (SEER) database. A total of 6,996 cases met our inclusion criteria (Fig. 1), which included 4,599 patients with tumor size 3 to 4 cm, and 2,397 patients with tumor size 4 to 5 cm. The baseline characteristics of patients in each group were showed in Table 1. Of the total number of patients, 5,876 were 60 years old or older, and 1,120 patients were less than 60 years old. Patients included 3,650 males and 3,346 females. Cancer locations of cases were categorized as left lobe (2,944) and right lobe (4,052). Histological types were divided into adenocarcinoma (3,545) and nonadenocarcinoma (3,451). NSCLC tumors were also divided according to the degree of differentiation, into well and moderately differentiated (3,763), poor and undifferentiated (2,844) or tumors with other degree of differentiation (389). Sites of primary tumors were categorized as upper lobe (4,118), middle lobe (335), lower lobe (2,343), main bronchus (46), overlapping lesion of lung (103) and other (51). Based on the number of regional nodes examined, patients were divided into 5 groups: none (517), 1 to 30 nodes (5,727), 31 to 60 nodes (159), 61 to 90 nodes (8) and other (585). The scopes of regional lymph node surgery were categorized as none (443), 1 to 3 scopes (1,004), ≥4 scopes (5,201) and other scopes (348). Patients underwent various surgical procedures, including wedge resection (570), segmental resection (190), lobectomy (5,936), pneumonectomy (202), and other surgical modalities (98).

**Table 1:** Baseline characteristics of patients with non-small-cell lung cancer.

Characteristics	Number of Patients			
	All patients (n = 6996)	Tumor size 3–4 cm (n = 4599)	Tumor size 4–5 cm (n = 2397)	P
Age	—	—	—	0.362
<60 y	1120	723	397	
≥60 y	5876	3876	2000	
Sex				0.037
Male	3650	2358	1292	
Female	3346	2241	1105	
Race				0.905
White	5914	3886	2028	
Nonwhite	1082	713	369	
Location				0.435
Left	2944	1920	1024	
Right	4052	2679	1373	
Histology				<0.001
Adenocarcinoma	3545	2416	1129	
Nonadenocarcinoma	3451	2183	1268	
Differential degree				<0.001
Well or Moderate	3763	2561	1202	
Poor or Undifferentiated	2844	1803	1041	
Unknown	389	235	154	
Primary site				<0.001
Up	4118	2744	1374	
Middle	335	241	94	
Low	2343	1508	835	
Main bronchus	46	19	27	
Overlapping lesion of lung	103	57	46	
Other	51	30	21	
Regional nodes examined	—	—	—	0.149
None	517	345	172	
1 to 30	5727	3780	1947	
31 to 60	159	94	65	
61 to 90	8	3	5	
Other	585	377	208	
Scope of regional LN surgery	—	—	—	0.014
None	443	303	140	
1 to 3	1004	700	304	
> = 4	5201	3371	1830	
Other	348	225	123	
Surgery modality				<0.001
Wedge resection	570	434	136	
Segmental resection	190	136	54	
Lobectomy	5936	3872	2064	
Pneumonectomy	202	107	95	
Other	98	50	48	

All p values were two sides and less than 0.05 were considered significant.

### Effect of tumor size on patient prognosis and survival

**Figure 2:**
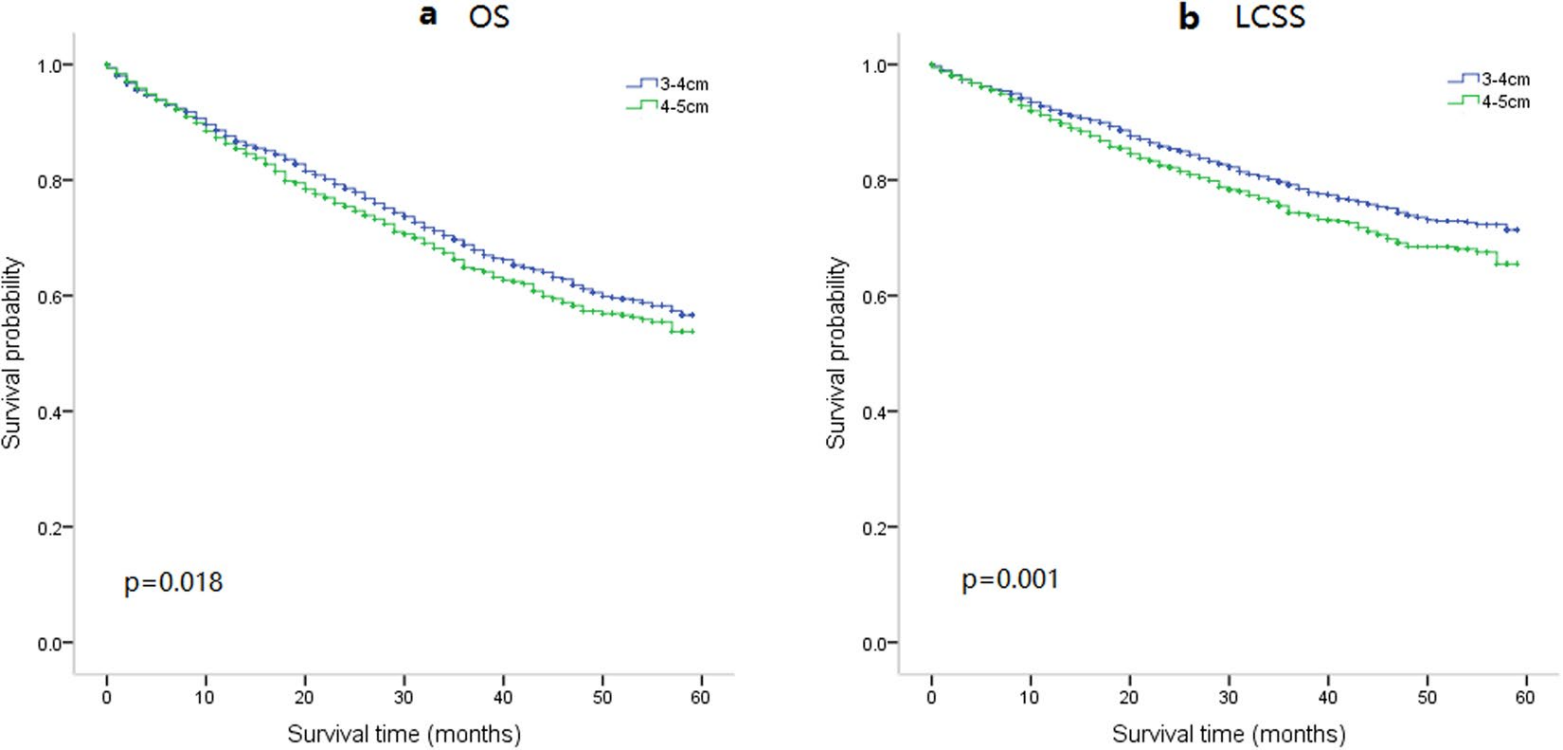
Kaplan–Meier survival curves to analyze overall survival and lung cancer-specific survival between patients with tumors of 3 to 4 centimeters and 4 to 5 centimeters two groups. (**a**) Kaplan–Meier survival curves for overall survival (OS) between 4,599 patients with tumors of 3 to 4 cm and 2,397 patients with tumors of 4 to 5 cm. The long-rank value (Mantel–Cox) is 5.622, p = 0.018. (**b**) Kaplan–Meier survival curves for lung cancer-specific survival (LCSS) between 4.599 patients with tumors of 3 to 4 cm and 2,397 patients with tumors of 4 to 5 cm. The long-rank value (Mantel–Cox) is 11.992, p = 0.001. All p values were two sides and less than 0.05 were considered significant.

Selected cases were classified into stages T2a (3 to 4 cm) and T2b (4 to 5 cm) groups. We used Kaplan–Meier survival curves to analyze overall survival (OS) and lung cancer specific survival (LCSS) between these two groups, as shown in Fig. 2. There were significant differences between the two groups, in both OS (p = 0.018) and LCSS (p = 0.001).

Results of survival analyses by univariate and multivariate Cox regression, for OS and LCSS, in NSCLC are shown in Tables 2 and 3. In the univariate Cox regression analysis, older age, male, white race, nonadenocarcinoma histology, 4 to 5 cm tumor size, poor or undifferentiated, other primary site, no regional nodes examined, no scope of regional lymph node surgery and other surgical modalities predicted worse OS and LCSS. However, there were no significant differences in either OS or LCSS based on cancer locations and tumor primary sites. In multivariate analysis, data were adjusted for age, sex, race, tumor histology, tumor size, degree of differentiation, regional nodes examined, scope of regional lymph node surgery and surgical modality. Results showed that tumor size was an independent predictor of both OS and LCSS. The new stage T2a (3 to 4 cm tumor size) patients had a better prognosis than new stage T2b (4 to 5 cm tumor size) patients, regarding both OS and LCSS, as listed in Tables 2 and 3.

**Table 2:** Univariate Cox Regression of prognostic factors in NSCLC.

Variables	Number of patients			
	OS		LCSS	
	HR(95% CI)	P	HR(95% CI)	P
Age		<0.001		<0.001
<60 y	1		1	
≥60 y	1.606(1.389–1.857)		1.409(1.186–1.674)	
Sex		<0.001		<0.001
Male	1		1	
Female	0.624(0.567–0.686)		0.654(0.581–0.736)	
Race		0.007		0.015
White	1		1	
Nonwhite	0.828(0.722–0.949)		0.808(0.681–0.959)	
Location		0.549		0.715
Left	1		1	
Right	1.029(0.937–1.130)		1.022(0.909–1.148)	
Histology		<0.001		<0.001
Adenocarcinoma	1		1	
Nonadenocarcinoma	1.359(1.238–1.492)		1.248(1.111–1.400)	
Tumor size		0.018		0.001
3–4 cm	1		1	
4–5 cm	1.122(1.020–1.235)		1.231(1.094–1.385)	
Differential degree				
Well or Moderate	1	<0.001	1	<0.001
Poor or Undifferentiated	1.354(1.232–1.488)	<0.001	1.476(1.311–1.661)	<0.001
Unknown	0.943(0.753–1.181)	0.61	1.074(0.819–1.409)	0.604
Primary site				
Up	1	0.058	1	0.082
Middle	0.814(0.639–1.036)	0.095	0.739(0.539–1.013)	0.06
Low	1.051(0.951–1.160)	0.329	1.054(0.932–1.193)	0.4
Main bronchus	1.767(1.135–2.750)	0.012	1.645(0.929–2.911)	0.088
Overlapping lesion of lung	1.080(0.742–1.572)	0.687	1.376(0.908–2.085)	0.132
Other	0.957(0.554–1.654)	0.875	1.028(0.533–1.984)	0.935
Regional nodes examined				
None	1	<0.001	1	<0.001
1 to 30	0.514(0.445–0.594)	<0.001	0.470(0.395–0.559)	<0.001
31 to 60	0.611(0.435–0.860)	0.005	0.518(0.334–0.803)	0.003
61 to 90	0.422(0.059–3.011)	0.39	0	0.885
Other	0.533(0.432–0.656)	<0.001	0.491(0.381–0.634)	<0.001
Scope of regional LN surgery				
None	1	<0.001	1	<0.001
1 to 3	0.537(0.447–0.645)	<0.001	0.515(0.412–0.644)	<0.001
>=4	0.481(0.414–0.560)	<0.001	0.444(0.369–0.533)	<0.001
Other	0.452(0.349–0.585)	<0.001	0.400(0.289–0.553)	<0.001
Surgery modality				
Wedge resection	1	<0.001	1	<0.001
Segmental resection	0.925(0.703–1.217)	0.577	0.803(0.569–1.133)	0.212
Lobectomy	0.593(0.511–0.688)	<0.001	0.534(0.447–0.638)	<0.001
Pneumonectomy	0.838(0.638–1.103)	0.207	0.845(0.611–1.171)	0.312
Other	1.420(1.044–1.932)	0.025	1.424(0.987–2.054)	0.059

All p values were two sides and less than 0.05 were considered significant. NSCLC = non-small-cell lung cancer, OS = overall survival, LCSS = lung cancer specific survival, HR = hazard ratio, CI = confidence interval.

**Table 3:** Multivariate Cox Regression of prognostic factors in NSCLC.

Variables	Number of patients			
	OS		LCSS	
	HR(95% CI)	P	HR(95% CI)	P
Age		<0.001		0.001
<60 y	1		1	
≥60 y	1.511(1.305–1.750)		1.345(1.130–1.601)	
Sex		<0.001		<0.001
Male	1		1	
Female	0.662(0.601–0.729)		0.688(0.611–0.775)	
Race		0.05		0.048
White	1		1	
Nonwhite	0.872(0.760–1.000)		0.841(0.707–0.999)	
Histology		<0.001		0.224
Adenocarcinoma	1		1	
Nonadenocarcinoma	1.199(1.089–1.320)		1.077(0.956–1.213)	
Tumor size		0.043		0.001
3–4 cm	1		1	
4–5 cm	1.105(1.003–1.218)		1.217(1.080–1.372)	
Differential degree				
Well or Moderate	1	<0.001	1	<0.001
Poor or Undifferentiated	1.289(1.170–1.420)	<0.001	1.427(1.264–1.610)	<0.001
Unknown	0.836(0.665–1.051)	0.125	0.928(0.704–1.223)	0.594
Regional nodes examined				
None	1	0.528	1	0.343
1 to 30	0.772(0.533–1.117)	0.17	0.653(0.421–1.013)	0.057
31 to 60	0.880(0.543–1.428)	0.605	0.702(0.386–1.277)	0.246
61 to 90	0.562(0.076–4.130)	0.571	0	0.888
Other	0.847(0.570–1.257)	0.409	0.746(0.465–1.196)	0.224
Scope of regional LN surgery				
None	1	0.163	1	0.182
1 to 3	0.850(0.566–1.277)	0.434	1.002(0.616–1.630)	0.994
> = 4	0.774(0.520–1.152)	0.207	0.879(0.545–1.417)	0.596
Other	0.669(0.431–1.038)	0.073	0 0.704(0.412–1.203)	0.2
Surgery modality				
Wedge resection	1	<0.001	1	<0.001
Segmental resection	1.015(0.768–1.340)	0.918	0.882(0.622–1.252)	0.483
Lobectomy	0.768(0.642–0.919)	0.004	0 0.694(0.558–0.862)	0.001
Pneumonectomy	1.042(0.776–1.399)	0.784	1.053(0.740–1.499)	0.775
Other	1.428(1.045–1.951)	0.025	1.393(0.960–2.021)	0.081

All p values were two sides and less than 0.05 were considered significant. NSCLC = non-small-cell lung cancer, OS = overall survival, LCSS = lung cancer specific survival, HR = hazard ratio, CI = confidence interval.

### Choice of surgical modality for new stages T2a and T2b

**Figure 3:**
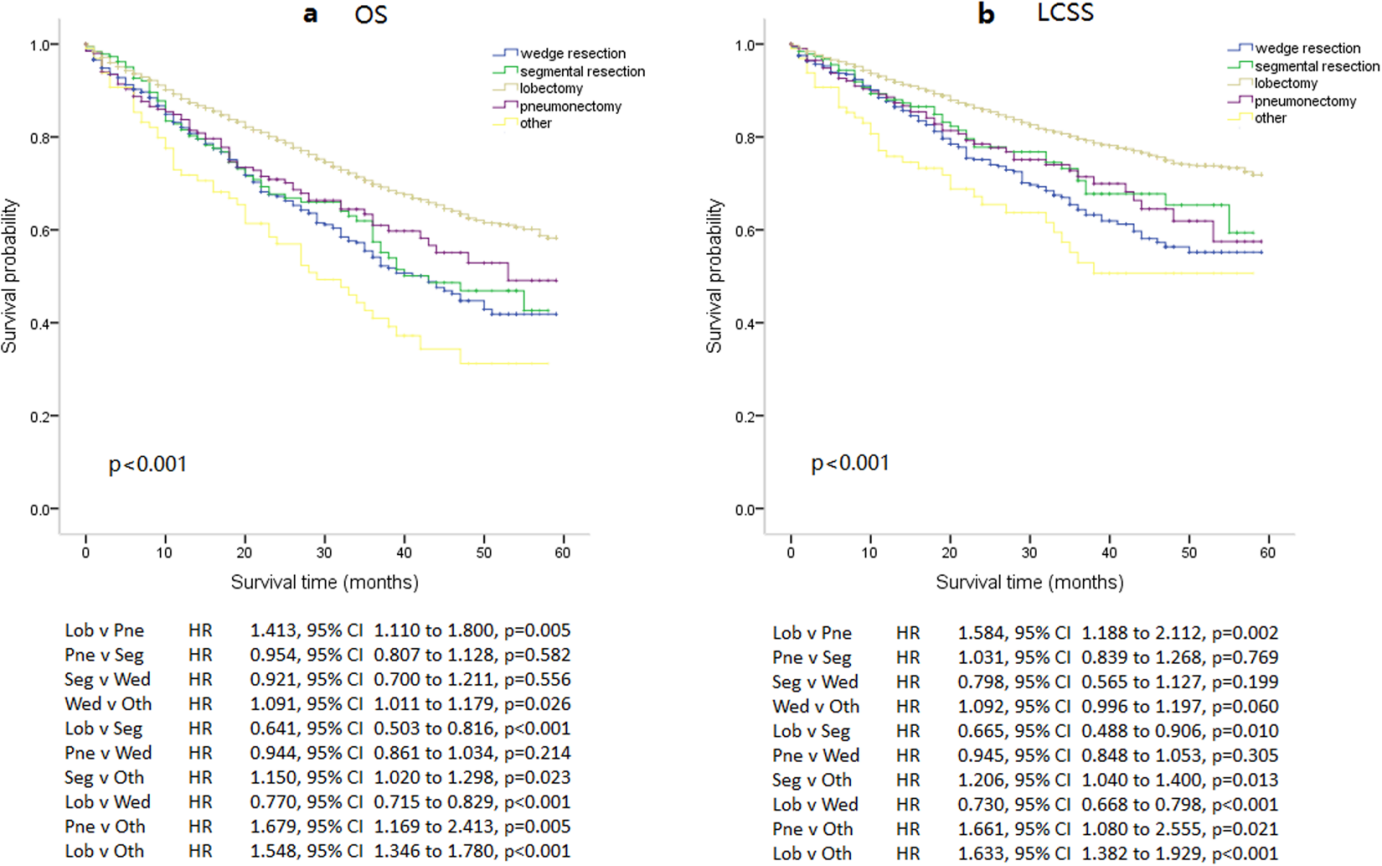
Kaplan–Meier survival curves for overall survival (OS) and lung cancer-specific survival (LCSS) between five different surgery modalities groups: lobectomy group (5,936), segmental resection group (190), wedge resection group (570), pneumonectomy group (202), and other surgery modalities group (98). (**a**) Kaplan–Meier survival curves for OS, the long-rank value (Mantel–Cox) is 96.302, p < 0.001. (**b**) Kaplan–Meier survival curves for LCSS, the long-rank value (Mantel–Cox) is 88.710, p < 0.001. All p values were two sides and less than 0.05 were considered significant. Lob = lobectomy group, Pne = pneumonectomy group, Seg = segmental resection group, Wed = wedge resection group, Oth = other surgery modalities group, HR = hazard ratio, CI = confidence interval.

Selected patients were classified into 5 groups according to the surgical procedure chosen for their treatment, which included wedge resection, segmental resection, lobectomy, pneumonectomy and other surgical modalities. Kaplan–Meier survival curves and the log-rank test were used to analyze OS and LCSS between these 5 groups, as shown in Fig. 3. There were significant changes in both OS (p < 0.001) and LCSS (p < 0.001) of these 5 groups of patients. Patients treated by lobectomy had a longer survival, observed in both OS and LCSS. However, there were no significant differences among wedge resection, segmental resection and pneumonectomy, in either OS or LCSS.

**Figure 4:**
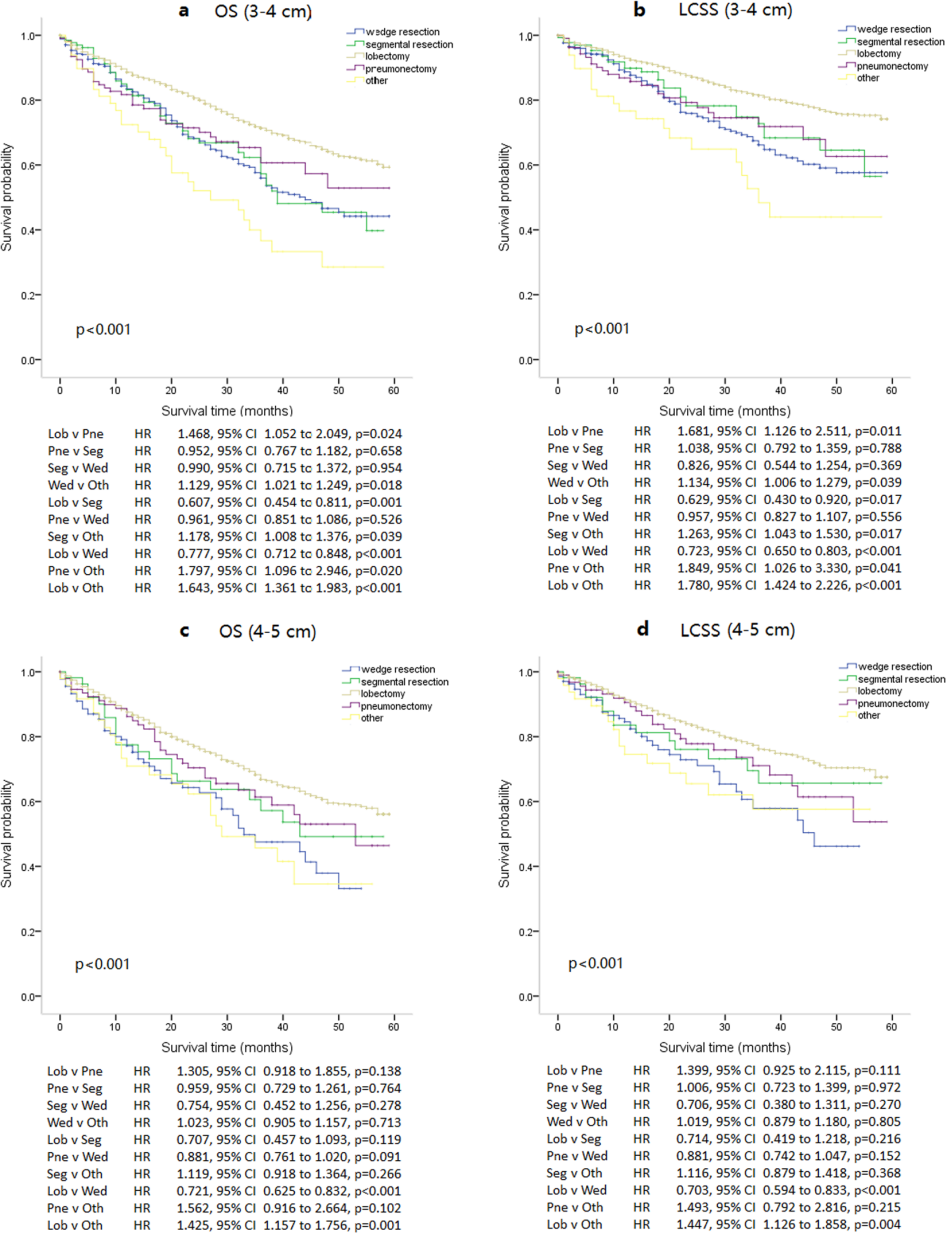
Kaplan–Meier survival curves of two tumor size groups for overall survival (OS) and lung cancer-specific survival (LCSS) between five different surgery modalities groups: lobectomy group, segmental resection group, wedge resection group, pneumonectomy group, and other surgery modalities group.(**a**) Kaplan–Meier survival curves for OS of patients with tumors of 3 to 4 cm, the long-rank value (Mantel–Cox) is 68.512, p < 0.001. (**b**) Kaplan–Meier survival curves for LCSS of patients with tumors of 3 to 4 cm, the long-rank value (Mantel–Cox) is 68.020, p < 0.001. (**c**) Kaplan–Meier survival curves for OS of patients with tumors of 4 to 5 cm, the long-rank value (Mantel–Cox) is 33.219, p < 0.001. (**d**) Kaplan–Meier survival curves for LCSS of patients with tumors of 4 to 5 cm, the long-rank value (Mantel–Cox) is 26.635, p < 0.001. All p values were two sides and less than 0.05 were considered significant. Lob = lobectomy group, Pne = pneumonectomy group, Seg = segmental resection group, Wed = wedge resection group, Oth = other surgery modalities group, HR = hazard ratio, CI = confidence interval.

Subgroup analysis was performed to compare patient survival between new stages T2a and T2b (Fig. 4 **)**. The outcomes of different surgical modalities were different between these two groups. For new stage T2a patients, lobectomy resulted in a significantly better prognosis, regarding both OS and LCSS. Lobectomy was the most appropriate surgical treatment modality. There were no significant differences in patient survival among wedge resection, segmental resection and pneumonectomy. Patients treated with other surgical modalities had the worst prognosis. However, the situation was different for new stage T2b patients. Though lobectomy patients had a significantly better prognosis than wedge resection patients, there were no significant differences in survival among pneumonectomy, segmental resection and lobectomy patients. Consequently it was difficult to choose the most appropriate surgical approach for new T2b stage patients among wedge resection, segmental resection, lobectomy and pneumonectomy.

## Discussion

The present study focused on the new stages T2a and T2b in the 8^th^ TNM staging system. Complementing the changes in TNM staging^8^, the appropriate surgical modalities also changed. Results indicated that lobectomy was the most appropriate surgical treatment modality for new stage T2a (3 to 4 cm tumor size) patients. However, for new stage T2b (4 to 5 cm tumor size) patients, the choice of surgical modality among lobectomy, wedge resection, segmental resection and pneumonectomy, depended on actual specific situations. We concluded that lobectomy was more appropriate than wedge resection for new stage T2b patients according to the prognosis analysis.

The major revisions of the 8th edition TNM classification for lung cancer staging, by the IASLC International Staging Project, are intended to improve the survival of lung cancer patients by increasing the precision of tumor staging^18^. In the 8th edition TNM classification, the T descriptor has been changed from the 7th edition TNM classification^13,14,19^. The newly introduced T stages raise interesting questions, leading us to considered that the appropriate surgical treatment modalities might have changed accordingly. There is currently no consensus regarding the most suitable treatment choice for operable tumors between 3 cm and 5 cm^20–22^.

It has been reported that surgery is the most appropriate treatment method for patients with early stage NSCLC^7,17^. However, the most suitable surgical modality for early stage NSCLC is yet to be determined^23^. Some associated studies have indicated that lobectomy was the first choice of stage I NSCLC lung cancer. He *et al* ^17^. indicated that lobectomy was better than sublobar resection for NSCLC patients with tumors measuring 0 to 2 cm. Moreover, for patients with tumors of 1 to 2 cm, segmentectomy was more appropriate than wedge resection. However, for NSCLC patients with tumor size 0 to 1 cm, the choice between segmentectomy and wedge resection depended on specific situations. Nevertheless, there are still some disputes about the most suitable treatment choice for these patients, especially in the elderly^24–26^. For instance, Razi *et al*. showed that sublobar resection was not inferior to lobectomy for elderly T1aN0M0 NSCLC patients. Though there were many studies exploring the association between surgical methods and TNM staging^24,27^, none have focused on NSCLC patients with 3 to 5 cm tumor size. This is the first study to explore the most appropriate surgical modalities for the new stages T2a and T2b in the 8th edition TNM staging system, based on a large population selected from the SEER database. Results indicated that, for stage T2a NSCLC patients, lobectomy had a significantly better prognosis regarding both OS and LCSS. The most appropriate surgical treatment modality for these patients was lobectomy. However, among wedge resection, segmental resection and pneumonectomy, there were no significant differences in either OS or LCSS. Patients with other surgical modalities had the worst prognosis. The situation was different for 4 to 5 cm tumor size NSCLC patients. Though lobectomy patients had a significantly better prognosis than wedge resection patients, there were no significant differences in survival among lobectomy, pneumonectomy and segmental resection patients. Although it is difficult to choose the best surgical modality for stage T2b patients among lobectomy, wedge resection, segmental resection and pneumonectomy, we could conclude that lobectomy was better than wedge resection surgery. These findings could be used as guidelines in choosing the best surgical modality for NSCLC patients with tumors measuring 3 to 5 cm.

We compared our analysis results by SEER database to the analysis results by IASLC database^8,19^, and identified some limitations to our study. Firstly, our paper is a retrospective study. Although we performed some measures to control bias, there are still some biases that could not be balanced. Secondly, the choice of surgical modality for NSCLC patients is not only determined by the size of the tumor, but also by the general performance of patients, pulmonary function, the primary site of lung cancer, *etc*. However, the SEER database lacks related information. Furthermore, although the SEER database is a population based database that includes cancer stage data and patient survival information in the United States^12^, it is not without problems. For example, the detailed classification of lung adenocarcinoma is not standardized and straightforward. Furthermore, the database lacks some factors related to lung cancer, such as smoking data, general patient performance, R0 margin resection, *etc*. Also, there is no information regarding the use of adjuvant chemotherapy and radiotherapy. Fortunately, adjuvant chemotherapy and radiotherapy are not very important in the treatment of early stage lung cancer patients.

This study showed that it was appropriate to separate 3 to 5 cm tumor size to new stages, namely, T2a (3 to 4 cm) and T2b (4 to 5 cm), due to their different prognosis. Lobectomy is the most appropriate surgical treatment modality for new T2a stage patients. The choice of surgical modality for patients that cannot tolerate lobectomy, among wedge resection, segmental resection and pneumonectomy, depends on surgical skills and patients’ situation. For new T2b stage patients, the best choice of surgical modality among lobectomy, wedge resection, segmental resection and pneumonectomy, is unclear and depends on the actual specific situation. Nevertheless, we conclude that lobectomy is better than wedge resection, according to the prognosis analysis in this stage.

## Patients and Methods

### Ethics statement

We obtained permission to access the SEER research data files using the reference number 11971-Nov2015. The data released by the SEER database do not require informed patient consent, and our study was approved by the Ethical Committee and Institutional Review Board of Shandong Provincial Hospital Affiliated to Shandong University. The methods were performed in accordance with the approved guidelines.

### Data source

We collected all our data about NSCLC from the SEER database, which includes collected clinical pathological data about multiple types of cancer cases in the United States, representing close to one-third of the US population. This database is updated every year to cover the new follow-up information and bring novel cases into the database. We selected all patients with histologically confirmed NSCLC, with 3 to 5 cm tumor size, diagnosed between 2009 and 2013. We confirm that all our methods and experimental protocols are according to relevant requests.

### Study population

The inclusion criteria were as follows: the patients of SEER database who were histological diagnosed as NSCLC between 2009 and 2013 were selected using the SEER Stat software. We only collected malignant behavior cases whose tumor sizes were between 3–5 cm, with no lymph node invasion and no metastasis. Moreover, we only selected patients whose tumor locations were left or right and only patients who had received surgical treatment.

The exclusion criteria were as follows: small cell lung cancer patients were excluded. Patients whose N stage and M stage were N1, NX, NA, or M1, MX, MA were all excluded. Patients with no information regarding surgical treatment were excluded. Patients whose diagnosis was established clinically, rather than by histology, were excluded.

Flow sheet of the number of eligible patients included in the study was shown in Fig. 1.

### Study variables

Patient information, including year of diagnosis, demographic information (age, race and sex), tumor size, location of lung cancer, histological type of the tumor, degree of differentiation, site of primary tumor, examination of regional nodes, scope of regional lymph node surgery, surgical modality, survival information and cause of death, was obtained from the SEER database. For the baseline characteristics analysis of patients with NSCLC, all cases were classified into two groups, according to the tumor size. In univariate and multivariate analysis, age, sex, race, location of lung cancer, histology, degree of differentiation, site of primary tumor, examination of regional nodes, scope of regional lymph node surgery, and surgical modality, were all classified as categorical variables. Age was classified into two groups, namely, less than 60 years old and more or equal to 60 years old. Cancer location was categorized as left and right lobe. Histological type of the tumor was divided into adenocarcinoma and nonadenocarcinoma. Cancer degrees of differentiation included well or moderate degree, poor or undifferentiated degree and unknown degree of differentiation. The sites of the primary tumors were categorized as upper, middle, lower, main bronchus, overlapping lesion of lung and other (including lung with NOS). Regional nodes examined were categorized into 5 groups, namely, no regional nodes examined, 1 to 30 regional nodes examined, 30 to 60 regional nodes examined, 60 to 90 regional nodes examined, and other group (including, no regional lymph nodes removed but aspiration of regional lymph nodes performed, and number of lymph nodes examined unknown or not stated). Scope of regional lymph node surgery was divided into none (no regional lymph nodes removed), 1 to 3 (1, regional lymph nodes removed; 2, intrapulmonary, ipsilateral hilar or ipsilateral peribronchial nodes; 3, ipsilateral mediastinal or subcarinal nodes), ≥4 (4, combination of 2 and 3; 5, contralateral mediastinal, contralateral hilar, ipsilateral or contralateral scalene or supraclavicular nodes; 6, combination of 5 with 2 or 3), other scope (unknown). Surgical modalities used in the treatment of selected patients included wedge resection, segmental resection, lobectomy, pneumonectomy and other surgical methods (including surgery NOS, excision NOS, laser excision, bronchial sleeve resection ONLY, resection of lung NOS).

### Endpoints

OS and LCSS were used to evaluate the prognosis of NSCLC patients^11^. OS was determined as the time from the diagnosis of lung cancer to the death of the patient, independent from the cause of death. LCSS was defined as the time from the diagnosis of lung cancer to the death of the patient, only when this was a direct consequence of lung cancer. For OS, patients who were alive on December 31, 2013, were defined as censored data. For LCSS, patient death due to causes other than lung cancer or alive at the end of the follow-up period, were all recognized as censored data.

### Statistics

SPSS version 20 was used for the statistical analysis. To examine the association between each baseline clinicopathological variable and tumor size, the Pearson ${\chi }^{2}$. coefficient was used. Univariate Cox proportional hazards regression and multivariate Cox proportional hazards regression were used to assess the association between survival information (including OS and LCSS, expressed as p value, hazards regression, 95% confidence interval) and the other variables. Kaplan–Meier survival curve analysis and log-rank test were used to compare the survival information (OS or LCSS) of the newly defined T2a (3–4 cm) and T2b (4–5 cm) stages. All p values were two-sided and values lower than 0.05 were considered significant.

### Data availability

Datasets generated and analyzed in this study are available in the SEER data repository, [https://seer.cancer.gov/].
